# Finding Biomarkers in Antioxidant Molecular Mechanisms for Ensuring Food Safety of Bivalves Threatened by Marine Pollution

**DOI:** 10.3390/antiox11020369

**Published:** 2022-02-11

**Authors:** María López-Pedrouso, José M. Lorenzo, Zulema Varela, J. Ángel Fernández, Daniel Franco

**Affiliations:** 1 Departamento de Zooloxía, Xenética e Antropoloxía Física, Universidade de Santiago de Compostela, 15872 Santiago de Compostela, Spain; mariadolores.lopez@usc.es; 2 Centro Tecnolóxico da Carne de Galicia, Rúa Galicia No. 4, Parque Tecnolóxico de Galicia, San Cibrao das Viñas, 32900 Ourense, Spain; jmlorenzo@ceteca.net; 3 Área de Tecnoloxía dos Alimentos, Facultade de Ciencias, Universidade de Vigo, 32004 Ourense, Spain; 4 CRETUS, Ecology Unit, Department of Functional Biology, Universidade de Santiago de Compostela, 15872 Santiago de Compostela, Spain; zulema.varela@usc.es (Z.V.); jangel.fernandez@usc.es (J.Á.F.)

**Keywords:** aquaculture products, emerging pollutants, omic technologies, stress proteins, antioxidant enzymes, oxidative stress

## Abstract

Aquaculture production as an important source of protein for our diet is sure to continue in the coming years. However, marine pollution will also likely give rise to serious problems for the food safety of molluscs. Seafood is widely recognized for its high nutritional value in our diet, leading to major health benefits. However, the threat of marine pollution including heavy metals, persistent organic pollutants and other emerging pollutants is of ever-growing importance and seafood safety may not be guaranteed. New approaches for the search of biomarkers would help us to monitor pollutants and move towards a more global point of view; protocols for the aquaculture industry would also be improved. Rapid and accurate detection of food safety problems in bivalves could be carried out easily by protein biomarkers. Hence, proteomic technologies could be considered as a useful tool for the discovery of protein biomarkers as a first step to improve the protocols of seafood safety. It has been demonstrated that marine pollutants are altering the bivalve proteome, affecting many biological processes and molecular functions. The main response mechanism of bivalves in a polluted marine environment is based on the antioxidant defense system against oxidative stress. All these proteomic data provided from the literature suggest that alterations in oxidative stress due to marine pollution are closely linked to robust and confident biomarkers for seafood safety.

## 1. Introduction

Aquaculture offers an improved opportunity for cultivating and harvesting marine food including fish, crustaceans, mollusc and aquatic plants through efficient large-scale production. For this reason, aquaculture production has been rising by around 6.24% in terms of metric tonnes each year from 1994 to 2020 [1]. Another major advantage of the aquaculture industry over fisheries is that aquaculture by-products are being processed to extract bioactive compounds, resulting in higher sustainability [2]. Within the aquaculture industry, farmed bivalve species grown in estuarine and coastal environments such as lagoons and coastal ponds have a significant economic value. Among worldwide mollusc species, the most important in terms of their large production (17.1 million tonnes in 2016) are the following: 28% Cupped oysters nei (*Crassostrea* spp.), 25% Japanese carpet shell (*Ruditapes philippinarum*), 11% Scallops nei (*Pectinidae*), 7% Marine molluscs nei (*Mollusca*), 6% Sea mussels nei (*Mytilidae*) and 5% Constricted tagelus (*Sinonovacula constricta*) [3].

**Figure 1:**
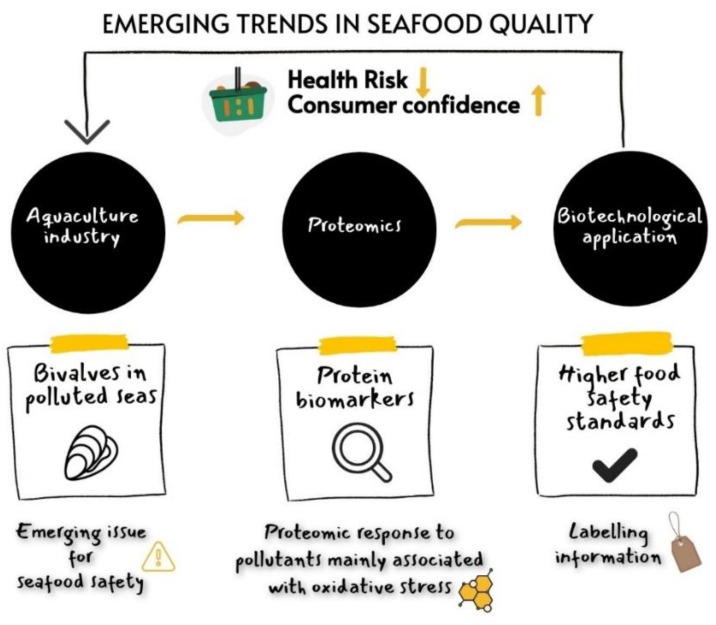
Emerging challenges in seafood quality.

Food safety remains a major concern for consumers around the world. It has such importance that one of the pillars of the EU Green Deal is Farm to Fork, a strategy to comprehensively address the challenges of sustainable food systems by recognizing the links between healthy people, healthy societies and a healthy planet. Ensuring and preventing failures in regard to food quality and safety are key targets of the food industry to avoid public health problems. Within food safety, microbiological outbreaks and chemical risks due to pollutants are considered as the main food industrial challenges in the 21st century [4]. On this line, environmental pollution and its adverse consequences in the agricultural, livestock and fishery sectors are being extensively researched. The consumption of polluted marine organisms is studied from a food toxicological point of view because certain chemical compounds pose great danger for human health. Specifically, edible bivalve molluscs could be hazardous due to their high filtering capacity along with exceptional resistance to pollutants, which can result in a high level of accumulation in them. Consequently, the pollution environmental threat is of paramount importance to measure both its effect on food safety as well as in the productive and nutritional quality of bivalves; the health status of the bivalve should be monitored to create a safer environment for consumers (Figure 1).

Aquatic pollution due to anthropological activities and their impact on ecosystems is currently a huge concern for society. As reported by World Wild Fund (WWF) in 2017, pollution in coastal and marine environments has become more relevant in the last few years [5]. Additionally, this is a complex issue because of the wide variety of pollutants and their interactions in the aquatic environment. Certainly, the variety of pollutants is rapidly growing. The majority come from agricultural activities (mainly ammonium and nitrate ions included in fertilizers, pesticides and agrochemicals), from domestic and municipal wastes, and sewage sludge (pathogens, organic substances, heavy metals and other trace elements). Many other sources of pollution are described such as oils spills or even aquaculture activities [6]. Another large group are chemicals such as persistent organic pollutants, pharmaceuticals and personal care products, veterinary medicines, as well as endocrine-disrupting chemicals and nanomaterials [7]. Their presence in the open sea turns them into bioactive and toxic compounds for living organisms at low doses [8]. Beyond these compounds, the concern of emerging contaminants such as microplastics is growing due to their potential toxicity and persistence in marine organisms; thus, they should be assessed in more detail [9]. These contaminants, including microplastics [10], nanoparticles [11] and organic chemicals [12], also accumulate in bivalves, but information on this issue is scarce. Additionally, there are other environmental problems associated with climate global changes, which increase the resulting abiotic stress due to chemical pollution. For instance, environmental parameters such as temperature, salinity, dissolved oxygen and pH are closely associated with climatic change [13]. This complex picture has led to the need to detect and assess the impact of pollution, specifically low amounts of an increasingly complex mixture of pollutants, on environmental quality. Biological changes can occur in response to pollutants or natural stressors associated with the climate changes mentioned above. As the pollutant concentrations are not informative of the potential toxic effects on organisms—providing only information on the bioaccumulation of themselves—new approaches are necessary.

**Figure 2:**
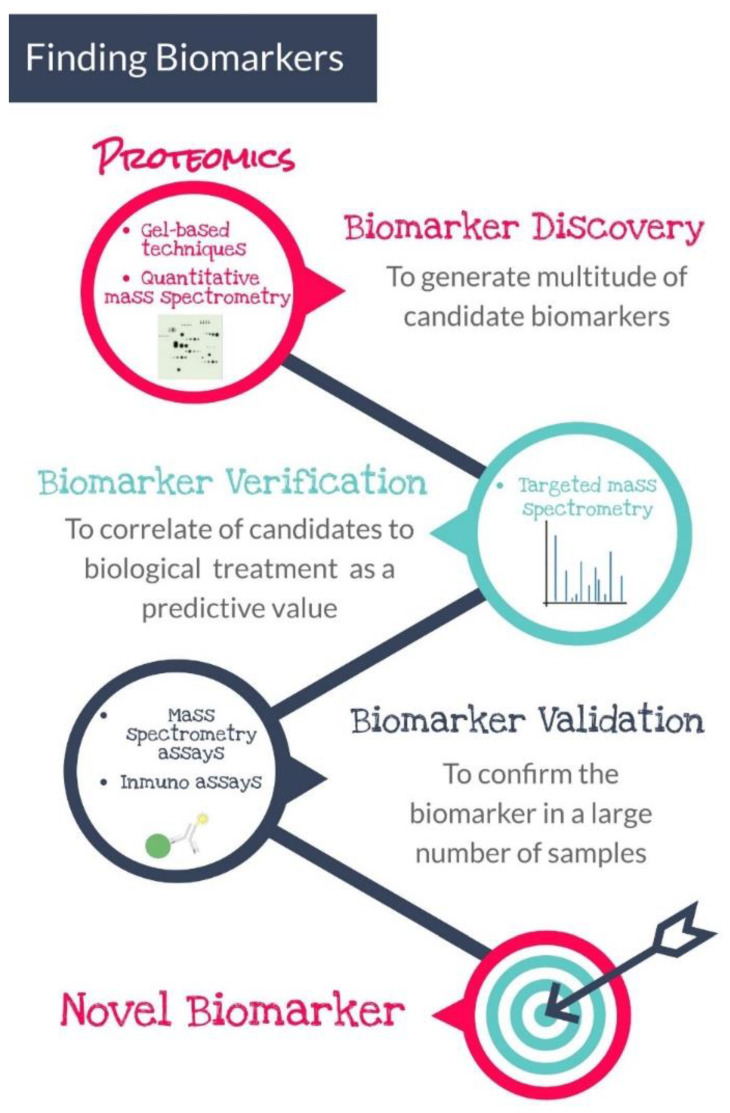
Workflow commonly used for finding protein biomarkers in the field of food technology science. Biomarker discovery is the first step of the process of development, and further validation and verification of the data should be carried out to ensure the novel biomarker is applicable in different scenarios.

Foodomics, including genomics, transcriptomics, proteomics and peptidomics, focuses on identifying new bioactive components associated with food quality and safety attributes [14]. A high-throughput proteomic approach allows the searching of protein biomarkers with high sensitivity and specificity as well as a thorough understanding of the biological process. This protein information could open up relevant perspectives for the food industry. The detecting and quantification of suitable protein biomarkers using available analytical instrumentation could be particularly effective in the field of food safety. However, technological development of analysis for new protein biomarkers implies a great number of steps from biomarker discovery to the validation phase, as shown in Figure 2. This novel approach allows us to understand the response of the organisms to the pollutants at a molecular level, as well as the search of biomarkers, which leads to immunoassay, targeted mass spectrometry, etc. This new methodology could reach a higher sensibility, multiple pollutants could be measured with only a few biomarkers, or it could be used for validating other results. 

This review aims to highlight relevant and innovative applications of proteomics to search biomarkers of marine bivalves in antioxidant molecular mechanisms, ensuring food safety. This will be an emerging challenge within the marine contamination field in future years.

## 2. Farming Marine Bivalves in Polluted Seawater

### 2.1. Benefits of Marine Bivalves in the Human Diet: Nutritional Quality

Seafood is recognized for its important health benefits and high nutritional value in our diet. More specifically, it is well known that molluscan shellfish contain high amounts of protein and other substances such as vitamin B12, choline, Se, Fe and Zn, as well as being low in calories and fat content. They are also characterized by a high glycogen content because it is a primary metabolic reserve [15]. Their high protein content is of interest as an alternative to other traditional sources and, like other marine organisms, their fatty acid profile is very interesting from a nutritional point of view. Indeed, despite being a low-fat food, bivalve lipids have beneficial effects on human health as a source of natural omega-3 LC-PUFA, particularly eicosapentaenoic acid (20:5n-3, EPA) and docosahexaenoic acid (22:6n-3, DHA). However, lipid content and the fatty acid profile could be modified by environmental conditions such as food availability and physiological conditions [16]. Beyond this intrinsic nutritional profile, this food has an enormous number of bioactive compounds with antiviral, anti-inflammatory, and antimicrobial activity [17].

### 2.2. Emerging Health Risk of Eating Marine Bivalves

**Figure 3:**
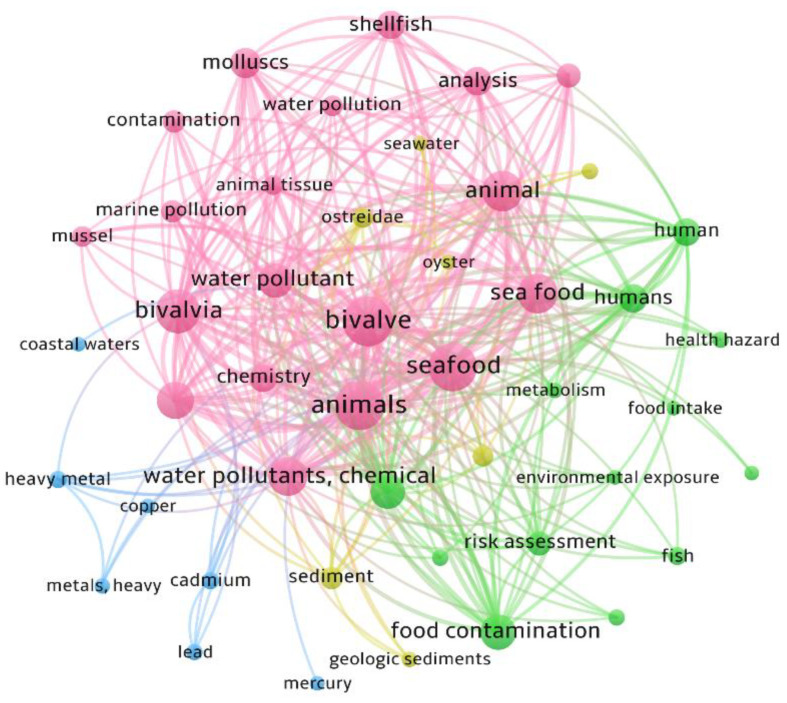
Map based on bibliographic data using co-occurrence of keywords among the documents collected from the Scopus database using the keywords “seafood”, “bivalves”, “pollution” and “contamination” in the abstracts and titles as search parameters. VOSviewer was employed to display the overlay visualization of keywords relationships. Only those with a minimum of 25 citations were included and the 30 most cited keywords were considered. According to the analysis, the documents were categorized into three clusters (**red**, **blue** and **green**).

There is great concern about the detrimental effects of contaminated marine food consumption on human health. The health-promoting potential of marine molluscs offers an opportunity for the food and nutraceutical industry [17]. However, a polluted aquatic environment could threaten this market. The balance between human health risk and benefits is a complex task that still needs to be tackled by the food industry [18]. A brief bibliometric analysis of documents collected from the Scopus database using the keywords “seafood”, “bivalves”, “pollution” and “contamination” in the abstracts and titles as search criteria identified over 124 documents published mostly in the environmental science field (107 documents); these studies were published in the period between 2012 and 2021. In Figure 3, these documents are analyzed, using VOSviewer software (https://www.vosviewer.com/ accessed on 3/9/2020) to depict them visually. The analysis of the keywords included in each document illustrated a growing concern over human health (e.g., health hazard, food contamination, risk assessment) in edible marine organisms (e.g., mussel, ostreidae, molluscs, shellfish), suggesting that environmental science is paying more attention to food safety. The literature review indicated that the most common chemical substances studied are dichlorodiphenyltrichloroethane (DDT), dioxins, endosulfan, heavy metals, persistent organic pollutants, pesticides, polybrominated diphenyl ethers, polychlorinated biphenyls (PCBs) and polycyclic aromatic hydrocarbons (PAHs), among others. Out of these, 74 documents focused on the most important heavy metal pollutants (i.e., Cd, Pb and Hg). Regarding organic pollutants, PAHs were thoroughly studied in 57 documents. Shellfish exposure to bio-accumulative pollutants and PCBs, as well as emerging contaminants such as microplastics (18 documents), have also been investigated in recent years. 

### 2.3. Marine Pollution as a Threat to Food Safety of Bivalves

#### 2.3.1. Heavy Metals and Metalloids

**Figure 4:**
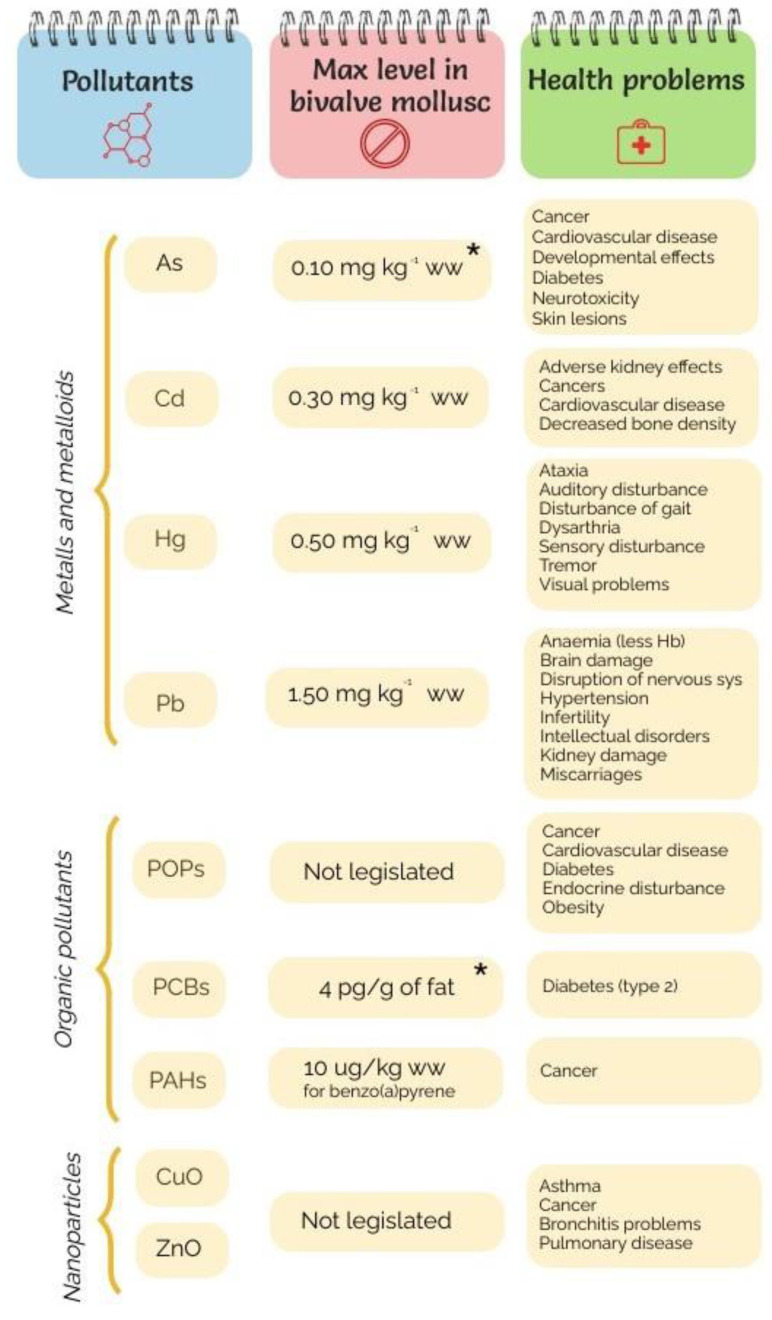
Main health problems caused by marine pollutants pose a major threat to sustainable aquaculture and food safety. The maximum permitted levels in bivalve molluscs (except for PCBs, which is in fishery products as indicated by *) are regulated by European Commission (EC) No. 1881/2006 of 2006. The maximum level of arsenic in foodstuffs is according to CODEX, S. (1995).

Major health problems caused by marine pollutants of seafood pose a serious risk for the global population; there are minimum levels set by regulations in most cases, as summarized in Figure 4. Metal contamination has been more thoroughly studied than other pollutants and their health effects have been determined in detail, as can be seen in Figure 4. For many years, metal contamination has been widely studied. Particularly, As, Cr, Cd, Pb and Hg are quite dangerous, causing mutagenic, carcinogenic and genotoxic effects on human health [19]. The bivalve tissue damage may lead to choking and poor appearance, resulting in economic losses. Malformations of mussel shells could also be correlated with the concentration of Pb, Hg and Sn, and even interactions with other factors related to water quality, such as nitrogen compounds, phosphate, turbidity, salinity and pH, have been demonstrated [20].

As is a metalloid that mainly arises from anthropogenic sources since it can be used as a color agent in textile manufacturing, insecticide or rodent killer. Water and food contaminated with As in its inorganic and other more toxic forms have been reported around the world. Specifically, As poisoning could cause the development of skin problems, cancers, cardiovascular and diabetic diseases or neurotoxic problems [19,21]. In this sense, inorganic As concentrations in blue mussels (*Mytilus edulis*) were detected above 0.53 mgKg^−1^ in Norwegian Fiords. For a person over 70 Kg, a blue mussel intake portion of 200 g with this As concentration leads to a 10% excess of tolerable weekly intake [22]. Other reported values of As concentrations ranged from 4 to 40 mg kg^−1^ in *Mytilus galloprovincialis* [23,24,25], leading to increased danger of poisoning. 

Regarding Cd, it is currently used as a stabilizer in plastics and semiconductors in Ni-Cd batteries and other photovoltaic devices and other industrial and agricultural activities that lead to anthropogenic emissions. The World Health Organization [26] gave us a warning about Cd’s toxic effects on human health, particularly on the kidneys, skeletal system and respiratory system, posing a major public health concern. In this sense, this metal was classified as an important human carcinogenic and the European Food Safety Authority (EFSA) established a provisional tolerable weekly intake of Cd of 0.0025 mg kg body weight^−1^ in 2012 [27,28]. In most cases, this intake dose of Cd is rarely exceeded through mussel consumption, but seafood contributes significantly to increase the level of Cd intake [29]. Cd levels at a concentration of 0.13 mg kg^−1^ in *Mytilus galloprovincialis* have been reported from Catalonia, Spain [30], while this value was seven times higher in specimens of *Mytilus chilensis,* reaching a concentration of 0.95 mg kg^−1^ from the Chilean coast [31].

Concerning Pb, coal combustion, leaded fuels and production of pyrometallurgical nonferrous metals contribute to marine pollution. Hence, Pb emissions into the marine environment should be monitored. It should be noted that the health impact associated with Pb poisoning is huge and well-known [32]. Pb has an affinity for bone, replacing the calcium that is crucial for skeleton growth. In this sense, children are particularly sensitive to toxic effects accumulating in growing bones. In the case of acute effects, gastrointestinal disturbances, neurological effects, hepatic and renal damage and hypertension could occur [33]. In this sense, a high concentration of Pb ranging from 0.1 to 17.15 μg g^−1^ in different mussel organs (*Perna perna*) has been reported. Indeed, the concentration detected was high, even in cases of water quality being healthy according to European norms [34]. Beyond this, salinity conditions, temperature and other seasonal parameters are affecting the impact of metals such as Pb in *Mytilus galloprovincialis* through detoxification activity [35]. 

Another potential hazard of seafood consumption is Hg, mainly as methylmercury (CH_3_Hg)^+^, which is highly bioavailable and toxic. The main sources of mercury come from industrial activities, mining and fossil fuel burning. Subsequently, mercury could be transformed into other forms depending on the oxidation or reduction conditions of the aquatic environment. In 1956, it was reported for the first time that methylmercury poisoning of the Japanese population was due to the consumption of seafood from Minamata Bay [36]. The consumption of fish with high concentrations of methylmercury could cause serious health problems, being considered as neuro and cardio-toxic compounds, and should be avoided by consumers wherever possible [37]. In the case of Hg accumulation among bivalves, it has been proven that variability of species is also an added problem as well as other metals. The recommended mean weekly intake of Hg by a 60 kg person is under 0.01 mg kg^−1^; otherwise, serious health problems could occur [38]. 

For all these hazards, the level of metals in seafood consumed should be supervised because this problem is increasingly being observed for seafood safety beyond the current recommendations.

#### 2.3.2. Persistent Organic Pollutants

Evidence for the contamination of organic pollutants was also detected in the marine environment. The detrimental effects of persistent organic pollutants (POPs) such as PAHs, PCBs, polybrominated diphenyl ethers (PBDEs), dioxins, furans and chlorinated pesticides (OCPs) should be monitored. Overall, the POPs are specifically dangerous because they are bioaccumulated and biomagnified in living organisms [39]. From Figure 4, it can be inferred that organic pollution in connection with seafood safety is not controlled as well as metal pollution. Nevertheless, there is not a great deal of information regarding the toxicity level of organic compounds from bivalve intake as in the case of metal pollution. A possible explanation for this fact is based on the heterogeneity of organic molecules, which are difficult to analyze jointly. In general, several diseases such as diabetes, obesity, cancer, endocrine disturbance, and cardiovascular, among others, have been related to these POPs [40]. Moreover, the accumulation and quantification of POPs have not been sufficiently studied in the case of bivalves; consequently, we considered other seafood to illustrate the level of these organics in this section.

According to the CE (Commission Regulation 2011), one of the most hazardous pollutants present in insecticides and other industrial products, due to its high toxicity and lipophilic property, is dichlorodiphenyldichloroethylene (DDE) [41]. Indeed, it has been proven that high concentrations of DDE and other PCBs in fish consumption might be increasing levels of type 2 diabetes, suggesting a serious risk for the global population [42]. PCBs were detected in farmed and wild mussels (*Mytilus galloprovinciallis*), with a higher level in wild mussels due to these pollutants being more concentrated in marine sediments [43]. Nevertheless, molluscs do not yet pose a risk for consumers from the present data on concentration, along with the average consumption of bivalves [44,45,46].

Regarding PAHs, these organic compounds are composed of two or more aromatic rings, and they are mainly formed by incomplete combustions or pyrolysis of organic compounds during industrial activities. In this way, this particle pollution in seawater can also contribute to poisoning aquatic organisms, particularly mussels [47]. For this reason, the consumption of oil-contaminated seafood after oil spills poses a human health problem due to their carcinogenic potential. Based on existing literature reviewed by Farrington [48], the need to evaluate the human health risk of seafood intake polluted by PAHs is increasing. For example, in the Mediterranean Sea, the levels of sixteen PAHs in three fish species (*Sardina pilchardus, Solea solea and Donax trunculus*) were investigated, and resulted in a low risk of chronic systemic effects, although relative risk of cancer should be assessed in mollusc consumers [49].

### 2.4. Bivalve Microbial Contamination Exacerbated by Pollution

Another concern is the impact of pollutants on microbiological seafood safety and quality of edible bivalves, caused by the detrimental effects of pollution on the health status of the bivalve organism. Indeed, it is well-known that pathogens can more easily cause diseases in a weakened organism by adverse living conditions. As bivalve species are edible molluscs, routine controls of protein toxins, bacterial and fungal toxins, allergens, antinutrients compounds, foodborne pathogens and biopesticides must be carried out by government agencies [50]. These biological hazards, mainly pathogens and biotoxins, can easily lead to human illness because bivalve molluscs are often eaten raw. This problem is exacerbated due to mussels, oysters and clams being filter feeders and concentrating pathogens [51,52,53]. The most common pathogens belong to the norovirus family, which are single-stranded RNA viruses that cause acute gastroenteritis. However, other pathogens such as hepatitis E and hepatitis A virus are also linked to bivalve shellfish consumption. Bacteria of the genus Vibrio can also cause human infections in the case of raw or undercooked seafood. Nevertheless, bacterial and viral pathogens could be effectively eliminated by thermal treatments, high hydrostatic pressure, irradiation and others, but chemical compounds are a more serious problem [54]. However, this biological contamination could cause significant degradation of seafood quality. For the above reasons, knowledge on pollution accumulation is of great interest to ensure the safety of seafood and to improve its nutritional quality—which are both still unresolved. 

In sum, there is serious concern about direct human consumption of bivalve molluscs from polluted areas, as evidenced by recent studies that are trying to assess this problem at the international level [55,56]. As we have discussed so far, a consistent increase in aquatic pollution, as well as aquaculture production, poses a very serious health risk if it is not effectively controlled. The chemical contaminants in the environment are difficult to eliminate and the most efficient strategy is to avoid the intake of polluted seafood, meaning this involves a technological challenge. 

## 3. Proteomic Strategy in the Evaluation of Bivalves in Polluted Environments

For the reasons already given above, it is reasonable to expect that aquatic pollution is causing changes at the molecular level, including in transcriptional and translational steps. A protein biomarker seems to be appropriate for the evaluation of food safety in the case of seafood. In the past, environmental pollution has been related to free radical damage in biological systems and oxidative stress. Several molecular biomarkers of different chemical classes such as malondialdehyde or diene conjugated for lipid oxidation, others associated with DNA damage (hydroxylation of guanosine), antioxidant enzymes (glutathione peroxidase, superoxide dismutase, catalase), non-enzyme antioxidants (glutathione, vitamin E, ascorbate, betacarotene) as well as metallothioneins have been proposed to assess environmental pollution [57].

### 3.1. Proteomic Overview

#### Why Proteomics?

The recent development of high-throughput technologies (omics) allows us to generate knowledge at a large-scale molecular level. Thus, omics technologies such as genomics, transcriptomics, proteomics, and metabolomics together with statistical and bioinformatics tools offer enormous potential in the search of biomarkers at present. Among them, protein biomarkers are of particular interest for food authenticity, quality and safety in the field of food science, as corroborated by 864 papers published in the last five years (using Scopus and the keywords “proteomic” or “protein biomarker” and “food authenticity” and “food quality” and “food safety”). Within omics, proteomics is emerging as a new tool for unravelling critical aspects including biological, physiological and ecological traits of shellfish [2]. The main advantage is that the proteome is highly variable in response to diverse stimuli and environmental factors in contrast with the genome, which is more stable. The application of these high-throughput technologies to search for biomarkers of seafood contamination could improve the protocols used by aquaculture in the field of food safety. In this regard, marine environmental quality and the food safety of edible bivalves could be monitored and assessed by bioindicators because complex scenarios of contamination require greater effort to search for more robust and efficient biomarkers in aquatic bivalves [58]. Furthermore, in-depth knowledge of molecular pathways and a better understanding of alterations of organisms in response to aquatic pollution would be reached more easily in comparison with classical analytical methods of pollution assessment. On the other hand, the concentration of pollutants in the environment is not always correlated with the lethality of living organisms. The complexity of interactions between the environment and pollutants defines their bioavailability. Beyond this, the mechanisms of toxicity, bioaccumulation, degradation and transport of the pollutant are complex issues [59]. Different degrees of toxicity induced by pollutants and underlying molecular mechanisms can be analyzed from the perspective of protein expression [60,61]. In this sense, biomarkers would be an ecotoxicological approach to evaluate the physiological effects of pollutants in the organism. Proteomics as high-throughput protein tools can detect simultaneous changes in proteins underpinning a chain of events triggered by pollutant exposure [62].

### 3.2. Proteomic Tools

The classical technique of two-dimensional gel electrophoresis (2-DE) to separate proteins followed by mass spectrometry (MS), specifically MALDI-TOF/TOF, was the most used in the collected studies. Polyacrylamide gel electrophoresis can be employed to separate complex mixtures of proteins and, subsequently, the gel images can be analyzed to estimate the abundance of each protein from a different sample. The protein spots are excised and digested with trypsin, resulting in a specific proteolytic rupture. Thus, this peptide mass fingerprint is employed to identify the protein by MS. On the other hand, isotopic labelling strategies are used for biomarker discovery such as the isobaric tag for relative and absolute quantification (iTRAQ). This technique involves chemical labelling of resulting peptides from a digested protein mixture as the derivatization of peptides. After MS/MS fragmentation, signature ions and quantitative data are provided. In this case, labelled peptides from different bivalve organisms are mixed in a single sample and the resulting peptide mixture is separated by two-dimensional liquid chromatography (LC) and identified by MS, obtaining identification and quantification with high accuracy [63]. However, labelling approaches are more expensive and time-consuming. Currently, the most consistent and quantitatively accurate proteomics data come from liquid chromatography coupled to tandem mass spectrometry (LC-MS/MS) and data-independent acquisition (DIA) methods such as SWATH-MS [64], but their application in the environmental field is still scarce.

### 3.3. Bivalve Response to Polluted Environment: Oxidative Stress and the Search of Biomarkers

Among the 124 documents from Section 2.2, a collection of 24 studies were related to the main cellular alterations of contaminated seafood from a proteomic point of view (Table 1). The selection of this literature was performed using the database Scopus and the keywords “bivalve”, “mollusc”, “pollution”, “contamination” and “proteomic”, including a manual review. It should be pointed out that many biological processes and molecular functions of bivalves were affected by pollutants, indicating notorious toxicological effects (Table 1). Alterations in the cytoskeleton, cellular stress, energy metabolic, immune system and others were detected in bivalve species from polluted environments. From a proteomic point of view, these alterations are often described and significant changes in the bivalve proteome are detected, including within transplanted bivalves. 

The main response mechanism of the bivalve against the toxic environment is via antioxidant defenses. Oxygen is required by tissues to meet their energetic demands but, at the same time, free radicals could be generated, damaging molecules and cells, and altering the regulation of molecular pathways and levels of metabolites. Thus, oxidative stress is produced by an imbalance in the redox state of the cell. This imbalance between exogenous factors such as environmental pollution and endogenous antioxidant defenses in bivalves can be employed to estimate toxicological effects under stressful environmental conditions, with special emphasis on the oxidative stress mechanism. To date, reactive oxygen species are known to be responsible for a diversity of oxidative damages, but there are gaps in our knowledge on cellular damage, response mechanisms, repair processes, and disease etiology in bivalves. Therefore, in the following sections, we summarized the most recent advances in bivalve organisms in this sense.

#### 3.3.1. Chaperones and Heat Shock Proteins

A stress response to pollutants often triggers a cascade of events inducing the synthesis of a set of stress proteins including heat shock proteins that belong to the chaperone family. In general, chaperone proteins are involved in regulating protein folding, translocation reactions and protein aggregation. In this sense, rapid breakdown and reorganization of tissues are provoked by these proteins, which play an important role in several physiological processes [65]. According to Melwani et al., 2016, differentially abundant proteins such as tubulin-specific chaperone A, alpha-crystallin B chain, 78 kDa glucose-regulated protein, heat shock protein beta-1 and ferritin were detected in response to metal pollution from a comparative proteomic analysis in oysters (*Crassostrea hongkongensis*). Moreover, these molecular chaperones were related to the defensive system, including the antioxidative system. In line with this, ferritin was proposed as a protein biomarker of As and Fe contamination as well as cathepsin L. Other studies conducted in Sydney rock oysters (*Saccostrea glomerata*) agree with the aforementioned results. These oysters were exposed to several complex scenarios with a wide range of contaminants in the long term, resulting in heat shock protein-70 being highly expressed [66]. These findings suggest that bivalve organisms often use these proteins as an adaptative response to excess reactive oxygen species (ROS) production. Therefore, protein biomarkers related to heat shock proteins and other chaperones are remarkably reliable to study the effects of the contaminated environment in bivalves.

#### 3.3.2. Enzymatic Antioxidant Response and Alterations of Metabolism and Ubiquitin System

The ROS excess simultaneously triggers these biochemical events, activating critical enzymes such as catalase, superoxide dismutase and glutathione peroxidase. Thus, the effects of organic pollutants such as permethrin and anthracene were studied by Sellami et al. 2015 [83] on clam *Venerupis decussata*, observing a higher oxidative activity of these enzymes; in addition, these pollutants have a detrimental effect on shell structure. In the same way, another bivalve, *Saccostrea cucullate*, also suffered the effect of tributyltin (TBT) (concentrations ranging from 10 to 150 µgL^−1^), inducing defensive proteins including HSP-78, HSP-70, aldehyde dehydrogenase and catalase, among others. This set of proteins, after exposition to TBT, were significantly induced, because this organic pollutant provoked alterations in calcium homeostasis, suggesting a molecular mechanism to repair distortions in the shell calcification of *S. cucullate* [86]. 

In the case of Cd pollution, the level of ROS was monitored in *Tegillarca granosa* due to its strong ability to tolerate Cd. Iron-sulfur cluster scaffold protein (Nfu 1) was significantly upregulated under Cd detoxification and the SOD activity was inhibited [76]. Cd also interferes with DNA and protein metabolism in oyster via the generation of ROS, because Cd concentration above 6 µgL^−1^ caused abnormalities in protein folding and proteolysis, as well as DNA damage, inhibiting DNA repair [74]. These findings were consistent with other studies conducted with oysters *Crassostrea gigas* exposed to sanitary sewage for 14 days, despite there being a relevant number of proteins (25 out of 30) that could not be identified [69].

The glutathione system also contributes to protecting protein thiols. Glutathione peroxidase is a selenium-dependent hydroperoxides-reducing enzyme that can scavenge ROS by reducing H_2_O_2_ and fatty acid hydroperoxides. The glutathione peroxidase (GSH) is converted into the oxidized form (GSSG) through nicotinamide adenine dinucleotide phosphate (NADPH), which donates an electron. Therefore, the pentose phosphate pathway that generates NADPH could also be affected in response to environmental stress conditions [89]. A proteomic approach enabled the study of eleven putative GSHs by two-dimensional gel electrophoresis and MALDI-TOF/TOF in gills of *Mytilus galloprovincialis* and results on the proteomic profiles of the GSH were significantly different, reflecting the level of water pollution from the North of Portugal [71].

In other circumstances, aquatic pollution often disturbs the metabolic pathways of bivalve organisms. For example, the tricarboxylic acid cycle is used to generate NADH and FADH_2_ using acetyl CoA provided from pyruvate oxidation at the end of glycolysis. The reductive capacity of NADH and FADH_2_ is employed in the last steps of cellular respiration for the synthesis of ATP molecules in oxidative phosphorylation. The movement of electrons is harnessed in the synthesis of ATP, but a slight imbalance of electrons causes ROS [90]. Regarding metal contamination, studies in *Ruditapes philippinarum* revealed that a significant number of biological processes (tricarboxylic acid cycle, oxidative phosphorylation, fatty acid β-oxidation, stress resistance, apoptosis and mitochondrial fission) were affected [67]. In the case of the *Corbicula fluminea* subjected to pharmaceutical compounds, calreticulin, proliferating cell nuclear antigen, aldehyde dehydrogenase (ALDH), alcohol dehydrogenase, T-complex protein 1 and 6 phosphogluconate dehydrogenase were proposed as protein biomarkers. Metabolism (ALDH, alcohol dehydrogenase and 6 phosphogluconate dehydrogenase) proteins were affected in response to the complex contamination of psychiatric hospital chemical products [67]. 

Other biomarkers related to oxidative stress in bivalves are related to the ubiquitin-proteasome pathway, with a key role in the removal of altered unrepaired proteins [74]. The selective degradation of cellular proteins is marked by the ubiquitin molecule and, thus, influences cell cycle regulatory proteins. This would be of particular importance in tissue proteolysis in seafood. Increased synthesis of ubiquitin proteins under stressful conditions is because of the need to replace damaged proteins along the non-lysosomal pathway. Indeed, deep structural changes in proteins such as folding, sorting and degradation processes can be activated in response to stress. In the case of mussels, protein degradation is increased and cell defense proteins are decreased in a highly polluted environment. Moreover, warmer temperatures provoked an increase in heat shock stress response and ubiquitin-mediated degradation [70].

#### 3.3.3. Structural Proteins: Myosin and Actin 

It is also known that cellular structure is highly variable and time-dependent in terms of pollutant exposition. Therefore, proteins related to the structure and function of the cytoskeleton have been proposed as targets of oxidative stress in polluted environments. Myosin and actin are mainly associated with the development of cell movements, and the former is responsible for converting chemical energy from ATP into mechanical energy in muscle contraction and other movements, including cell division. On the contrary, actin is related to the movement of cells across a surface through actin polymerization, and migration, morphogenesis, cytokinesis, endocytosis and phagocytosis are the main cellular processes of the actin cytoskeleton. Both proteins (myosin and actin), along with tropomyosin in connection with shell properties, are often altered after stressful conditions in bivalves. For instance, the PAH benzo-α-pyrene in a concentration of 10 µgL^−1^ caused major disturbances in the cytoskeleton of oyster *Pinctada martensii*, but only metabolic biomarkers were proposed due to their major impact on osmotic regulation and energy metabolism [80]. Another proteomic study also highlighted the alteration of cell morphology (e.g., β-actin, tropomyosin, paramyosin, myosin regulatory light chain A and others), energy metabolism and stress response concerning the accumulation of PCBs (30 µgL^−1^) in *M. galloprovinciallis*, suggesting damage in cellular structures. In line with this, alterations at the histological level have been reported and are associated with proteomic changes in gills from *Mitylus galloprovinciallis* exposed to 30 µgL^−1^ of tetrabromobisphenol A. The histological alterations were mainly observed in the reduction in frontal cilia and lateral cilia, infiltration of hemocytes and a higher level of apoptotic nuclei [85]. 

**Figure 5:**
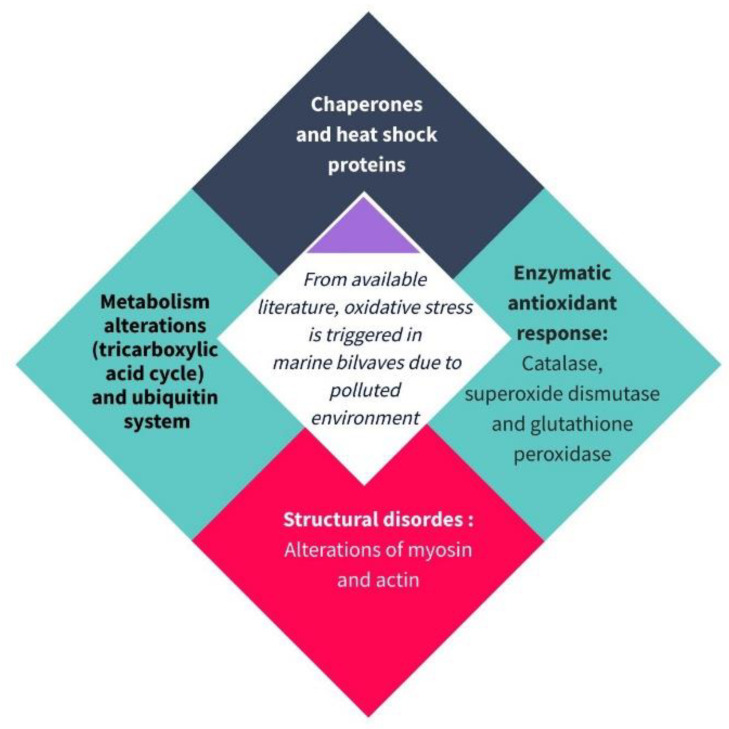
Pollution biomarkers of marine bivalves can be classified into four groups: chaperones and heat shock proteins, proteins involved in metabolism alterations and ubiquitin system, enzymes from the antioxidant response, and structural proteins.

All this information demonstrates that the molecular mechanisms of bivalve organisms due to aquatic pollution exposure could be analyzed to establish robust biomarkers. The proteomic approach could provide a robust and confident strategy to predict and characterize potential pollution hazards in seafood (Figure 5).

## 4. Final Remarks

Novel technological advances are required to detect a plethora of contaminants in bivalves, and proteomic technology is being developed for the discovery of protein biomarkers, addressing the most challenging tasks. In this regard, the marine pollution problem, which is a threat to the ecosystem and human health, is still not resolved. The thorough analysis of proteomes and the biological regulation of proteins allow us to gain new insights into many biological processes. It has been widely demonstrated that environmental pollutants affect the condition of the bivalves, causing economic losses in the aquaculture industry. Seafood safety and traceability as critical factors for consumers should be more strongly monitored to prevent the entrance of toxic pollutants into our diet. Moreover, emerging pollutants must be regulated and supervised to avoid any risk to public health. The lack of knowledge about hazardous pollutant accumulation and its damaging effects on edible bivalves may have serious consequences on human health soon, as epidemiological studies could demonstrate in the near future.

In addition to other biomarkers such as malondialdehyde or diene conjugated for lipid oxidation, others associated with DNA damage (hydroxylation of guanosine), antioxidant enzymes (glutathione peroxidase, superoxide dismutase, catalase), non-enzyme antioxidants (glutathione, vitamin E, ascorbate, beta carotene) as well as metallothioneins in this manuscript, we have reviewed the novel biomarkers for marine bivalves and classified them into four groups: chaperones and heat shock proteins, proteins involved in metabolism alterations and the ubiquitin system, enzymes from the antioxidant response, and structural proteins. These biomarkers could be used to determine contamination in molluscs to verify if the product is suitable for human consumption. However, this is the first step, hence more effort needs to be made to study the underlying mechanisms, biological pathways and interrelationship between the putative biomarkers involved. Further analyses should evaluate the robustness of biomarkers identified in this review using accurate methods and new populations of bivalves under different pollution scenarios.

## Figures and Tables

**Table 1:** A collection of papers focusing on cellular alterations of contaminated seafood using a proteomic strategy.

Pollutants of Food Safety Concern	Edible Bivalves	Proteomic Technology	Proteomic Alterations	References
**Field experiments (Unspecific contamination)**				
Study area: Field stations in the German Bight	*Mytilus sp.* Blue mussel	2-DE followed by MALDI-TOF/TOF	Cytoskeletal proteins Energy metabolism Stress proteins Protein relevant for metal detoxification	[58]
Study area: Sydney Harbor estuary	*Saccostrea glomerata* Oyster	2-DE followed by LC-MS/MS	Energy metabolism Cytoskeletal proteins	[66]
Study area contaminated with pharmaceutical compounds	*Corbicula fluminea*	2-DE followed by LC-MS/MS	Structural proteins Cellular functions Metabolism	[67]
Study area: Jiulongjiang Estuary in South China	*Crassostrea hongkongensis* Oyster	2-DE followed by MALDI-TOF/TOF	Cellular injuries Oxidative stress Immune stress	[68]
Area contaminated by a sanitary sewage	*Crassostrea gigas* Oyster	2-DE followed by MALDI-TOF/TOF	Structural protein Ubiquitination pathway conjugation Signal transduction Cell cycle/division	[69]
Study area: Le Havre, France	*Mytilus edulis* Blue mussels	LC-ESI-Q/TOF	Protein degradation Cell defense proteins Heat shock response	[70]
Study area: North of Portugal and Galicia in Spain	*Mytilus galloprovincialis* Mussel	2-DE followed by MALDI-TOF/TOF	Glutathione activity	[71]
Study area: estuaries from the south coast of Portugal	*Scrobicularia plana* Clam	2-DE followed by MALDI-TOF/TOF	Cellular responses to stress	[72]
**Metal contamination (Lab conditions)**				
As (III) and As (V)	*Mytilus galloprovincialis* Mussel	2-DE followed by MALDI-TOF/TOF	Cytoskeleton and cell structure Energy metabolism Cell development	[73]
Cd	*Crassostrea gigas* Oyster	iTRAQ	DNA and protein metabolism	[74]
Cd	*Ruditapes philippinarum* Clam	iTRAQ	Immune response Metabolism processes	[75]
Cd	*Tegillarca granosa* Clam	1-DE and Western blot analysis	Production of ROS SOD activity	[76]
Cd	*Ruditapes philippinarum* Clam	iTRAQ	Tricarboxylic acid cycle Oxidative phosphorylation Fatty acid β-oxidation Stress resistance and apoptosis Mitochondrial fission	[77]
Zn	*Crassostrea angulata* Oyster	nano-UPLC–MS/MS	Zn transport Ca transport Phosphate metabolism Immune regulation Oxidative stress responses Gene expression regulation Fat metabolism	[78]
Zn	*Crassostrea gigas* Pacific oyster	iTRAQ	Protein refolding Ubiquitin proteasome pathway Glutathione metabolism	[79]
**Organic compounds (Lab conditions)**				
Benzo(a)pyrene (PAH) and DDT	*Pinctada martensii * Pearl oyster	2-DE followed by MALDI-TOF/TOF	Energy metabolism Cytoskeleton Cell injury Oxidative stress Signal transduction	[80]
Crude oil	*Corbicula fluminea* Clam	nano-UPLC–MS/MS	Glanbia of clams: Synapse assembly System process Regulation of muscle contraction Gill of clams: Negative regulation of coagulation Signalling pathway Protein polymerization GTP metabolic process Tricarboxylic acid cycle Nucleoside diphosphate phosphorylation	[81]
Diesel fuel water-accommodated fraction	*Crassostrea brasiliana*	2-DE followed by MALDI-TOF/TOF	Xenobiotic biotransformation Cytoskeleton Processing and degradation of proteins Biosynthesis of glycolipids and glycoproteins Stress proteins Plasmalogen biosynthesis	[82]
Permethrin and anthracene	*Venerupis decussata* Clam	1-DE	Catalase activity Superoxide dismutase activity Glutathione transferase activity	[83]
PCBs	*Mytilus galloprovincialis* Mussels	2-DE and HPLC-ESI-MS/MS	Maintenance of cell morphology Energy metabolism Stress response	[84]
Tetrabromobisphenol (TBBPA)	*Mytilus galloprovincialis* Mussel	iTRAQ	Protein synthesis Cytoskeletal organization Defence mechanism Development	[85]
Tributyltin (TBT)	Saccostrea cucullate Hooded oyster	2-DE followed by LC-MS/MS	Defensive mechanisms Cytoskeletal organization Calcium homeostasis Energy metabolism Amino acid metabolism	[86]
**Emerging contaminants (Lab conditions)**				
Microplastics	*Mytilus edulis * blue mussels	nanoLC-MS/MS	Immune regulation Detoxification Metabolism Structural development	[87]
PVP/PEI coated silver nanoparticles	*Mytilus galloprovincialis* Mussel	2-DE followed by MALDI-TOF/TOF	Metabolism	[88]

## References

[1] Tacon A.G.J. (2020). Trends in Global Aquaculture and Aquafeed Production: 2000–2017. Rev. Fish. Sci. Aquac..

[2] López-Pedrouso M., Lorenzo J.M., Cantalapiedra J., Zapata C., Franco J.M., Franco D. (2020). Aquaculture and by-products: Challenges and opportunities in the use of alternative protein sources and bioactive compounds. Adv. Food Nutr. Res..

[3] FAO (2018). The State of World Fisheries and Aquaculture.

[4] Fung F., Wang H.S., Menon S. (2018). Food safety in the 21st century. Biomed. J..

[5] WWF WWF—World Wild Fund for Nature Marine Problems: Pollution. http://wwf.panda.org/about_our_earth/blue_planet/problems/pollution/.

[6] Islam M.S., Tanaka M. (2004). Impacts of pollution on coastal and marine ecosystems including coastal and marine fisheries and approach for management: A review and synthesis. Mar. Pollut. Bull..

[7] Kroon F.J., Berry K.L.E., Brinkman D.L., Kookana R., Leusch F.D.L., Melvin S.D., Neale P.A., Negri A.P., Puotinen M., Tsang J.J. (2020). Sources, presence and potential effects of contaminants of emerging concern in the marine environments of the Great Barrier Reef and Torres Strait, Australia. Sci. Total Environ..

[8] Brumovský M., Bečanová J., Kohoutek J., Borghini M., Nizzetto L. (2017). Contaminants of emerging concern in the open sea waters of the Western Mediterranean. Environ. Pollut..

[9] Zhang F., Man Y.B., Mo W.Y., Man K.Y., Wong M.H. (2020). Direct and indirect effects of microplastics on bivalves, with a focus on edible species: A mini-review. Crit. Rev. Environ. Sci. Technol..

[10] Ward J.E., Zhao S., Holohan B.A., Mladinich K.M., Griffin T.W., Wozniak J., Shumway S.E. (2019). Selective Ingestion and Egestion of Plastic Particles by the Blue Mussel (*Mytilus edulis*) and Eastern Oyster (*Crassostrea virginica*): Implications for Using Bivalves as Bioindicators of Microplastic Pollution. Environ. Sci. Technol..

[11] Zha S., Rong J., Guan X., Tang Y., Han Y., Liu G. (2019). Immunotoxicity of four nanoparticles to a marine bivalve species, *Tegillarca granosa*. J. Hazard. Mater..

[12] Rodil R., Villaverde-de-Sáa E., Cobas J., Quintana J.B., Cela R., Carro N. (2019). Legacy and emerging pollutants in marine bivalves from the Galician coast (NW Spain). Environ. Int..

[13] Munari M., Matozzo V., Chemello G., Riedl V., Pastore P., Badocco D., Marin M.G. (2019). Seawater acidification and emerging contaminants: A dangerous marriage for haemocytes of marine bivalves. Environ. Res..

[14] Munekata P.E.S., Pateiro M., López-Pedrouso M., Gagaoua M., Lorenzo J.M. (2020). Foodomics in meat quality. Curr. Opin. Food Sci..

[15] Anacleto P., Maulvault A.L., Bandarra N.M., Repolho T., Nunes M.L., Rosa R., Marques A. (2014). Effect of warming on protein, glycogen and fatty acid content of native and invasive clams. Food Res. Int..

[16] Tan K., Ma H., Li S., Zheng H. (2020). Bivalves as future source of sustainable natural omega-3 polyunsaturated fatty acids. Food Chem..

[17] Khan B.M., Liu Y. (2019). Marine Mollusks: Food with Benefits. Compr. Rev. Food Sci. Food Saf..

[18] Schlag A.K. (2010). Aquaculture: An emerging issue for public concern. J. Risk Res..

[19] Mishra S., Bharagava R.N., More N., Yadav A., Zainith S., Mani S., Chowdhary P. (2019). Heavy Metal Contamination: An Alarming Threat to Environment and Human Health. Environ. Biotechnol. Sustain. Futur..

[20] Riani E., Cordova M.R., Arifin Z. (2018). Heavy metal pollution and its relation to the malformation of green mussels cultured in Muara Kamal waters, Jakarta Bay, Indonesia. Mar. Pollut. Bull..

[21] Rasheed H., Slack R., Kay P. (2016). Human health risk assessment for arsenic: A critical review. Crit. Rev. Environ. Sci. Technol..

[22] Sloth J.J., Julshamn K. (2008). Survey of total and inorganic arsenic content in blue mussels (*Mytilus edulis* L.) from Norwegian fiords: Revelation of unusual high levels of inorganic arsenic. J. Agric. Food Chem..

[23] Argese E., Bettiol C., Rigo C., Bertini S., Colomban S., Ghetti P.F. (2005). Distribution of arsenic compounds in Mytilus galloprovincialis of the Venice lagoon (Italy). Sci. Total Environ..

[24] Belivermiş M., Kiliç Ö., Çotuk Y. (2016). Assessment of metal concentrations in indigenous and caged mussels (Mytilus galloprovincialis) on entire *Turkish coastline*. Chemosphere.

[25] Orescanin V., Lovrencic I., Mikelic L., Barisic D., Matasin Z., Lulic S., Pezelj D. (2006). Biomonitoring of heavy metals and arsenic on the east coast of the Middle Adriatic Sea using *Mytilus galloprovincialis*. Nucl. Instruments Methods Phys. Res. Sect. B Beam Interact. Mater. Atoms.

[26] WHO (2019). Exposure to cadmium: A major public health concern. Prev. Dis. Through Health Environ..

[27] World Health Organization (2011). Safety evaluation of certain food additives and contaminants in food. Prep. by Seventy-third Meet. Jt. FAO/WHO Expert Comm. Food Addit..

[28] EFSA (2012). Cadmium Dietary Exposure in the European Population.

[29] González N., Calderón J., Rúbies A., Timoner I., Castell V., Domingo J.L., Nadal M. (2019). Dietary intake of arsenic, cadmium, mercury and lead by the population of Catalonia, Spain: Analysis of the temporal trend. Food Chem. Toxicol..

[30] Falcó G., Llobet J.M., Bocio A., Domingo J.L. (2006). Daily intake of arsenic, cadmium, mercury, and lead by consumption of edible marine species. J. Agric. Food Chem..

[31] Houlbrque F., Hervé-Fernández P., Teyssié J.L., Oberhaënsli F., Boisson F., Jeffree R. (2011). Cooking makes cadmium contained in Chilean mussels less bioaccessible to humans. Food Chem..

[32] Jan A.T., Azam M., Siddiqui K., Ali A., Choi I., Haq Q.M.R. (2015). Heavy metals and human health: Mechanistic insight into toxicity and counter defense system of antioxidants. Int. J. Mol. Sci..

[33] Kralliedde L., Brooke N., Baker D., Karalliedde L., Murray V., Maynard R., Norman H.P. (2012). Toxicity of heavy metals and trace elements. Essentials of Toxicology for Health Protection: A Handbook for Field Professionals.

[34] Abderrahmani K., Boulahdid M., Bendou N., Aissani A. (2020). Seasonal distribution of cadmium, lead, nickel, and magnesium in several tissues of mussels from the *Algerian coasts*. Environ. Sci. Pollut. Res..

[35] Freitas R., Leite C., Pinto J., Costa M., Monteiro R., Henriques B., Di Martino F., Coppola F., Soares A.M.V.M., Solé M. (2019). The influence of temperature and salinity on the impacts of lead in *Mytilus galloprovincialis*. Chemosphere.

[36] Harada M. (1995). Minamata Disease: Methylmercury Poisoning in Japan Caused by Environmental Pollution. Crit. Rev. Toxicol..

[37] Rideout K., Kosatsky T. (2017). Fish for Dinner? Balancing Risks, Benefits, and Values in Formulating Food Consumption Advice. Risk Anal..

[38] Puspitasari R., Purbonegoro T., Suratno (2020). Health risk assessment of metal accumulated in marine bivalves from Semarang, Indonesia. AACL Bioflux.

[39] Vorkamp K., Rigét F.F. (2014). A review of new and current-use contaminants in the Arctic environment: Evidence of long-range transport and indications of bioaccumulation. Chemosphere.

[40] Alharbi O.M.L., Basheer A.A., Khattab R.A., Ali I. (2018). Health and environmental effects of persistent organic pollutants. J. Mol. Liq..

[41] EU (2011). Amending Regulation (EC) No 1881/2006 as regards maximum levels for dioxins, dioxin-like PCBs and non dioxin-like PCBs in foodstuffs. Off. J. Eur. Union.

[42] Marushka L., Batal M., David W., Schwartz H., Ing A., Fediuk K., Sharp D., Black A., Tikhonov C., Chan H.M. (2017). Association between fish consumption, dietary omega-3 fatty acids and persistent organic pollutants intake, and type 2 diabetes in 18 First Nations in Ontario, Canada. Environ. Res..

[43] Rodríguez-Hernández Á., Camacho M., Henríquez-Hernández L.A., Boada L.D., Valerón P.F., Zaccaroni A., Zumbado M., Almeida-González M., Rial-Berriel C., Luzardo O.P. (2017). Comparative study of the intake of toxic persistent and semi persistent pollutants through the consumption of fish and seafood from two modes of production (wild-caught and farmed). Sci. Total Environ..

[44] Santos L.L., Miranda D., Hatje V., Albergaria-Barbosa A.C.R., Leonel J. (2020). PCBs occurrence in marine bivalves and fish from Todos os Santos Bay, Bahia, Brazil. Mar. Pollut. Bull..

[45] Esposito M., Canzanella S., Lambiase S., Scaramuzzo A., La Nucara R., Bruno T., Picazio G., Colarusso G., Brunetti R., Gallo P. (2020). Organic pollutants (PCBs, PCDD/Fs, PAHs) and toxic metals in farmed mussels from the Gulf of Naples (Italy): Monitoring and human exposure. Reg. Stud. Mar. Sci..

[46] Chiesa L.M., Nobile M., Malandra R., Pessina D., Panseri S., Labella G.F., Arioli F. (2018). Food safety traits of mussels and clams: Distribution of PCBs, PBDEs, OCPs, PAHs and PFASs in sample from different areas using HRMS-Orbitrap® and modified QuEChERS extraction followed by GC-MS/MS. Food Addit. Contam.-Part A Chem. Anal. Control Expo. Risk Assess..

[47] Bansal V., Kim K.H. (2015). Review of PAH contamination in food products and their health hazards. Environ. Int..

[48] Farrington J.W. (2020). Need to update human health risk assessment protocols for polycyclic aromatic hydrocarbons in seafood after oil spills. Mar. Pollut. Bull..

[49] Ferrante M., Zanghì G., Cristaldi A., Copat C., Grasso A., Fiore M., Signorelli S.S., Zuccarello P., Oliveri Conti G. (2018). PAHs in seafood from the Mediterranean Sea: An exposure risk assessment. Food Chem. Toxicol..

[50] Jennings S., Stentiford G.D., Leocadio A.M., Jeffery K.R., Metcalfe J.D., Katsiadaki I., Auchterlonie N.A., Mangi S.C., Pinnegar J.K., Ellis T. (2016). Aquatic food security: Insights into challenges and solutions from an analysis of interactions between fisheries, aquaculture, food safety, human health, fish and human welfare, economy and environment. Fish Fish..

[51] López Cabo M., Romalde J.L., Simal-Gandara J., Gago Martínez A., Giráldez Fernández J., Bernárdez Costas M., del Hierro S.P., Pousa Ortega Á., Manaia C.M., Abreu Silva J. (2020). Identification of Emerging Hazards in Mussels by the Galician Emerging Food Safety Risks Network (RISEGAL). A First Approach. Foods.

[52] Gyawali P., Hewitt J. (2020). Faecal contamination in bivalve molluscan shellfish: Can the application of the microbial source tracking method minimise public health risks?. Curr. Opin. Environ. Sci. Health.

[53] Dubert J., Barja J.L., Romalde J.L. (2017). New insights into pathogenic vibrios affecting bivalves in hatcheries: Present and future prospects. Front. Microbiol..

[54] Martinez-Albores A., Lopez-Santamarina A., Rodriguez J.A., Ibarra I.S., Del Carmen Mondragón A., Miranda J.M., Lamas A., Cepeda A. (2020). Complementary methods to improve the depuration of Bivalves: A review. Foods.

[55] De Witte B., Devriese L., Bekaert K., Hoffman S., Vandermeersch G., Cooreman K., Robbens J. (2014). Quality assessment of the blue mussel (*Mytilus edulis*): Comparison between commercial and wild types. Mar. Pollut. Bull..

[56] Van Cauwenberghe L., Janssen C.R. (2014). Microplastics in bivalves cultured for human consumption. Environ. Pollut..

[57] Valavanidis A., Vlahogianni T., Dassenakis M., Scoullos M. (2006). Molecular biomarkers of oxidative stress in aquatic organisms in relation to toxic environmental pollutants. Ecotoxicol. Environ. Saf..

[58] Helmholz H., Lassen S., Ruhnau C., Pröfrock D., Erbslöh H.B., Prange A. (2015). Investigation on the proteome response of transplanted blue mussel (*Mytilus* sp.) during a long term exposure experiment at differently impacted field stations in the German Bight (North Sea). Mar. Environ. Res..

[59] Wang T., Wang L., Chen Q., Kalogerakis N., Ji R., Ma Y. (2020). Interactions between microplastics and organic pollutants: Effects on toxicity, bioaccumulation, degradation, and transport. Sci. Total Environ..

[60] Luque-Garcia J.L., Cabezas-Sanchez P., Camara C. (2011). Proteomics as a tool for examining the toxicity of heavy metals. TrAC-Trends Anal. Chem..

[61] Mirzajani F., Askari H., Hamzelou S., Schober Y., Römpp A., Ghassempour A., Spengler B. (2014). Proteomics study of silver nanoparticles toxicity on Oryza sativa L.. Ecotoxicol. Environ. Saf..

[62] Sanchez B.C., Ralston-Hooper K., Sepúlveda M.S. (2011). Review of recent proteomic applications in aquatic toxicology. Environ. Toxicol. Chem..

[63] Aggarwal K., Choe L.H., Lee K.H. (2006). Shotgun proteomics using the iTRAQ isobaric tags. Briefings Funct. Genom. Proteom..

[64] Ludwig C., Gillet L., Rosenberger G., Amon S., Collins B.C., Aebersold R. (2018). Data-independent acquisition-based SWATH - MS for quantitative proteomics: A tutorial. Mol. Syst. Biol..

[65] Sanders B.M. (1993). Stress proteins in aquatic organisms: An environmental perspective. Crit. Rev. Toxicol..

[66] Melwani A.R., Thompson E.L., Raftos D.A. (2016). Differential proteomic response of Sydney rock oysters (Saccostrea glomerata) to prolonged environmental stress. Aquat. Toxicol..

[67] Bebianno M.J., Sroda S., Gomes T., Chan P., Bonnafe E., Budzinski H., Geret F. (2016). Proteomic changes in Corbicula fluminea exposed to wastewater from a psychiatric hospital. Environ. Sci. Pollut. Res..

[68] Xu L., Ji C., Wu H., Tan Q., Wang W.X. (2016). A comparative proteomic study on the effects of metal pollution in oysters Crassostrea hongkongensis. Mar. Pollut. Bull..

[69] Flores-Nunes F., Gomes T., Company R., Moraes R.R.M., Sasaki S.T., Taniguchi S., Bicego M.C., Melo C.M.R., Bainy A.C.D., Bebianno M.J. (2015). Changes in protein expression of pacific oyster Crassostrea gigas exposed in situ to urban sewage. Environ. Sci. Pollut. Res..

[70] Péden R., Rocher B., Chan P., Vaudry D., Poret A., Olivier S., Le Foll F., Bultelle F. (2018). Highly polluted life history and acute heat stress, a hazardous mix for blue mussels. Mar. Pollut. Bull..

[71] Azevedo C.C., Guzmán-Guillén R., Martins J.C., Osório H., Vasconcelos V., da Fonseca R.R., Campos A. (2015). Proteomic profiling of gill GSTs in Mytilus galloprovincialis from the North of Portugal and Galicia evidences variations at protein isoform level with a possible relation with water quality. Mar. Environ. Res..

[72] González-Domínguez R., Santos H.M., Bebianno M.J., García-Barrera T., Gómez-Ariza J.L., Capelo J.L. (2016). Combined proteomic and metallomic analyses in Scrobicularia plana clams to assess environmental pollution of estuarine ecosystems. Mar. Pollut. Bull..

[73] Yu D., Ji C., Zhao J., Wu H. (2016). Proteomic and metabolomic analysis on the toxicological effects of As (III) and As (V) in juvenile mussel Mytilus galloprovincialis. Chemosphere.

[74] Meng J., Wang W., Li L., Yin Q., Zhang G. (2017). Cadmium effects on DNA and protein metabolism in oyster (*Crassostrea gigas*) revealed by proteomic analyses. Sci. Rep..

[75] Lu Z., Wang S., Shan X., Ji C., Wu H. (2019). Differential biological effects in two pedigrees of clam Ruditapes philippinarum exposed to cadmium using iTRAQ-based proteomics. Environ. Toxicol. Pharmacol..

[76] Qian G., Bao Y., Li C., Xie Q., Lu M., Lin Z. (2017). Nfu1 mediated ROS removal caused by Cd stress in *Tegillarca granosa*. Front. Physiol..

[77] Ji C., Lu Z., Xu L., Li F., Cong M., Shan X., Wu H. (2019). Evaluation of mitochondrial toxicity of cadmium in clam Ruditapes philippinarum using iTRAQ-based proteomics. Environ. Pollut..

[78] Luo L., Zhang Q., Kong X., Huang H., You W., Ke C. (2017). Differential effects of zinc exposure on male and female oysters (Crassostrea angulata) as revealed by label-free quantitative proteomics. Environ. Toxicol. Chem..

[79] Meng J., Wang W.X., Li L., Zhang G. (2017). Respiration disruption and detoxification at the protein expression levels in the Pacific oyster (*Crassostrea gigas*) under zinc exposure. Aquat. Toxicol..

[80] Chen H., Song Q., Diao X., Zhou H. (2016). Proteomic and metabolomic analysis on the toxicological effects of Benzo[a]pyrene in pearl oyster *Pinctada martensii*. Aquat. Toxicol..

[81] Miserazzi A., Perrigault M., Sow M., Gelber C., Ciret P., Lomenech A.M., Dalens J.M., Weber C., Le Floch S., Lacroix C. (2020). Proteome changes in muscles, ganglia, and gills in Corbicula fluminea clams exposed to crude oil: Relationship with behavioural disturbances. Aquat. Toxicol..

[82] Müller G., Lüchmann K.H., Razzera G., Toledo-Silva G., Bebianno M.J., Marques M.R.F., Bainy A.C.D. (2018). Proteomic response of gill microsomes of Crassostrea brasiliana exposed to diesel fuel water-accommodated fraction. Aquat. Toxicol..

[83] Sellami B., Khazri A., Mezni A., Louati H., Dellali M., Aissa P., Mahmoudi E., Beyrem H., Sheehan D. (2015). Effect of permethrin, anthracene and mixture exposure on shell components, enzymatic activities and proteins status in the Mediterranean clam Venerupis decussata. Aquat. Toxicol..

[84] Ambrosio L., Russo R., Salzano A.M., Paolo Serpe F., Ariano A., Tommasi N.D.E., Piaz F.D.A.L., Severino L. (2018). Accumulation of Polychlorinated Biphenyls in Mussels: A Proteomic Study. J. Food Prot..

[85] Ji C., Li F., Wang Q., Zhao J., Sun Z., Wu H. (2016). An integrated proteomic and metabolomic study on the gender-specific responses of mussels Mytilus galloprovincialis to tetrabromobisphenol A (TBBPA). Chemosphere.

[86] Khondee P., Srisomsap C., Chokchaichamnankit D., Svasti J., Simpson R.J., Kingtong S. (2016). Histopathological effect and stress response of mantle proteome following TBT exposure in the Hooded oyster *Saccostrea cucullata*. Environ. Pollut..

[87] Green D.S., Colgan T.J., Thompson R.C., Carolan J.C. (2019). Exposure to microplastics reduces attachment strength and alters the haemolymph proteome of blue mussels (*Mytilus edulis*). Environ. Pollut..

[88] Duroudier N., Cardoso C., Mehennaoui K., Mikolaczyk M., Schäfer J., Gutleb A.C., Giamberini L., Bebianno M.J., Bilbao E., Cajaraville M.P. (2019). Changes in protein expression in mussels Mytilus galloprovincialis dietarily exposed to PVP/PEI coated silver nanoparticles at different seasons. Aquat. Toxicol..

[89] Tomanek L. (2015). Proteomic responses to environmentally induced oxidative stress. J. Exp. Biol..

[90] Mailloux R.J., Bériault R., Lemire J., Singh R., Chénier D.R., Hamel R.D., Appanna V.D. (2007). The tricarboxylic acid cycle, an ancient metabolic network with a novel twist. PLoS ONE.

